# Effect of surface roughness of biomaterials on *Staphylococcus epidermidis* adhesion

**DOI:** 10.1186/s12866-014-0234-2

**Published:** 2014-09-02

**Authors:** Itaru Yoda, Hironobu Koseki, Masato Tomita, Takayuki Shida, Hidehiko Horiuchi, Hideyuki Sakoda, Makoto Osaki

**Affiliations:** Department of Orthopedic Surgery, Graduate School of Biomedical Sciences, Nagasaki University, 1-7-1, Sakamoto, Nagasaki, 852-8501 Japan; Division of Medical Devices, National Institute of Health Sciences, 1-18-1, Kamiyoga, Setagaya-ku, Tokyo, 158-8501 Japan

**Keywords:** Bacterial adhesion, Biomaterials, Roughness, *Staphylococcus epidermidis*

## Abstract

**Background:**

Implant-related infections are caused by adhesion of bacteria to the surface of biomaterials. In this *in vitro* research, we evaluated the ability of *Staphylococcus epidermidis* (ATCC35984) to adhere to the surface of solid biomaterials at different levels of roughness below 30 nm Ra and investigated the minimum level of roughness required to promote bacterial adhesion on five kinds of biomaterials: oxidized zirconium-niobium alloy (Oxinium), cobalt-chromium-molybdenum alloy (Co-Cr-Mo), titanium alloy (Ti-6Al-4 V), commercially pure titanium (Cp-Ti) and stainless steel (SUS316L), samples of which were categorized into a fine group and a coarse group according to surface roughness. The test specimens were physically analyzed and the viable bacterial density of the adhered bacteria was quantitatively determined (n = 20).

**Results:**

The amount of bacteria that adhered to the biomaterials in the coarse group was higher than those in the fine group. Oxinium, Ti-6Al-4 V and SUS316L in particular demonstrated statistically significant differences between the two groups (*P* < 0.05). Of the materials, the Co-Cr-Mo specimens exhibited significantly lower amounts of adhered bacteria than the Ti-6Al-4 V, Cp-Ti and SUS316L specimens in the fine group. Similarly, the Co-Cr-Mo specimens in the coarse group exhibited significantly lower values than the other four materials.

**Conclusions:**

These results suggest that minimum level of roughness affecting initial bacterial adherence activity differs according to the type of biomaterial used, and that even a surface roughness of below 30 nm Ra in Oxinium, Ti-6Al-4 V and SUS316L can promote bacterial adhesion. Relative hydrophobic Co-Cr-Mo surfaces were less susceptible to bacterial adherence.

## Background

In the field of orthopedic surgery, a variety of solid, artificial biomaterials with particular mechanical characteristics are frequently implanted in the human body for a wide range of purposes, including prostheses and trauma plates/nails. Implant-related infection is generally the most common serious complication of these biomaterials, which provide a site suitable for bacterial colonization [1]. When bacteria adhere to and proliferate on the biomaterial surface, they produce extracellular polymeric substances and form a biofilm. The biofilm envelopes the bacteria and protects them from the immune system and anti-bacterial agents. Moreover, the increased competence implied for biofilm-embedded bacteria, which results in a higher degree of horizontal transfer of genes including antibiotic resistance markers and the occurrence of persistent cells, may further enhance biofilm-related antibiotic resistance [2]. As a result, implant-related infections are extremely difficult to treat [3,4]. Although various methods of prevention have been devised, implant-related infections still occur today in 0.2–17.3% of cases of prosthetic orthopedic surgery [5-7]. Most infected implants, including total joint arthroplasty, necessitate removal or revision surgery. Bozic et al. reported that 14.8% of revision total hip arthroplasty and 25.2% of revision total knee arthroplasty performed in the USA during 2005-2006 were the result of infection [8,9]. Research into the problem of bacterial adhesion to biomaterials is therefore critically important from a clinical perspective.

Most implant-related infections are caused by the *Staphylococcus* genus [10-12]. *Staphylococcus epidermidis* (*S. epidermidis*), one of the most commonly isolated bacterial pathogens, is particularly capable of adhering to and aggregating on biomaterial surfaces and it can form biofilms on many different biomaterials [13,14]. The process of bacterial adherence is generally thought to be governed by van der Waals interactions, such that bacteria arrive at the surface of the artificial material by overcoming energy barriers through electrostatic repulsion, and then form colonies by way of initial reversible/irreversible adhesion [15,16]. Research has shown that polysaccharide intercellular adhesion-Polymeric N-acetyl-β-(1,6)-glucosamine (PIA/PNAG) play an important role not only in biofilm formation, but also in bacterial adhesion [14,17-20]. However, the exact mechanism of adhesion has yet to be determined because of the complex combination of numerous other factors related to the bacteria itself, the *in vivo* environment and the particular artificial material involved.

Biomaterials used for clinical purposes are strictly regulated through standards such as the International Organization for Standardization (ISO) and the American Society for Testing and Materials (ASTM). Biomaterials can be made of just a few kinds of standardized materials depending on their application, including titanium, stainless steel, and cobalt-chromium-molybdenum alloy (Co-Cr-Mo). Oxinium is an oxidized zirconium-niobium alloy commercialized as a new biomaterial in Japan in 2008. It is created by permeating a zirconium-niobium alloy with oxygen at a high temperature so that the surface is changed to a monoclinic zirconia ceramic with a depth of only 5 μm. As a result, Oxinium has the low abrasiveness on sliding surfaces of a ceramic, but has the strength of a metal. It also contains almost no toxic metals [21].

Steinberg et al. reported differences in bacterial adhesion to two different material surfaces, titanium and titanium alloy [22]. Recently, there have been a number of reports on the impact of the physical properties of the solid materials themselves on bacterial adhesion [23-31] and a particularly strong relationship between bacterial adhesion and surface roughness has been highlighted [28-31]. Rougher surfaces have a greater surface area and the depressions in the roughened surfaces can provide more favorable sites for colonization. Some previous reports have shown that bacterial adhesion *in vivo* is primarily determined by a surface roughness of Ra greater than 0.2 μm (200 nm) [32,33]. On the other hand, Lee et al reported in an *in vitro* study that the total amount of bacteria adherent on resin (Ra = 0.179 μm) was significantly higher than on titanium (Ra = 0.059 μm) or zirconia (Ra = 0.064 μm). However, they also demonstrated no significant difference between titanium and zirconia [34]. Öztürk et al indicated that the roughness difference of 3 to 12 nm Ra between as-polished and nitrogen ion-implanted Co-Cr-Mo contributes to bacterial adhesion behavior [35]. Thus, a general consensus has not been yet obtained in the literature regarding the minimum level of roughness required for bacterial adhesion. Furthermore, there are few studies that compare bacterial adherence capability on the same types of biomaterial that differ in surface roughness on the nanometer scale (Ra < 30 nm). To our knowledge, no other studies have been carried out to date that simultaneously evaluate the bacteriological characteristics of adhesion to five different types of material, including Oxinium.

In this *in vitro* study, we compared the adherence capability of *S. epidermidis* to biomaterials at different levels of roughness below 30 nm Ra and investigated the range of roughness that influences bacterial adhesion using five kinds of biomaterials that are actually used in clinical practice: Oxinium, Co-Cr-Mo, titanium alloy (Ti-6Al-4 V), commercially pure titanium (Cp-Ti) and stainless steel (SUS316L).

## Materials and methods

### Specimen preparation

We prepared circular specimens (12 mm in diameter, 6 mm thick) from Oxinium (ASTM F2384), cobalt-chromium-molybdenum alloy (Co-Cr-Mo) (ASTM F75 high carbon), titanium alloy (Ti-6Al-4 V) (ASTM F136), pure titanium (Cp-Ti) (ASTM F67) and stainless steel (SUS316L) (ASTM F138). Original materials were obtained from Smith & Nephew Orthopaedics Inc. (Memphis, TM, USA) and Kakushin Surgical Instruments Co. Ltd. (Shizuoka, Japan). The five types of test specimen were progressively polished using a basic lapping machine (Doctorlap ML-180SL, Maruto Co.Ltd., Tokyo, Japan) with polishing compounds, polishing cloths and diamond slurry (Maruto Instrument Co. Ltd., Tokyo, Japan; 1 μm particle diameter). We divided each biomaterial into two groups according to surface roughness: the fine group, which completed the abrasion step, and the coarse group, which did not perform the final abrasion step.

### Surface analysis

In order to observe the surface micro-structure, micrographs were obtained using a field emission scanning electron microscope (SEM: JSM 6610LV, JEOL, Tokyo, Japan). The micrographs were taken at two randomly chosen areas on each specimen (one in a central position and one at 1-1.5 mm in from the outer edge). The surface roughness of the specimen disks was measured by means of a 3D measuring laser microscope (OLS4000, Shimadzu, Tokyo, Japan) with a cut-off value (λc) of 80 μm at room temperature. To measure roughness, three readings were taken of each surface of two random samples, and the average roughness (Ra) was used to determine the roughness of the specimens. The initial contact angles of the surface of each specimen to deionized water (Milli-Q®, EMD Millipore, Billerica, MA, USA) were measured by the drop method using an automated contact angle measurement device (DSA30, Krüss GmbH, Hamburg, Germany) at room temperature. Prior to determining the contact angle, all specimens were equilibrated with ethanol. On each of three randomly selected specimens, three drops of deionized water (2 μL) were analyzed (twelve measurements in total per product), and the left and the right contact angles of each drop were averaged.

### Experimental design

*S. epidermidis* strain RP62A (American Type Culture Collection [ATCC] 35984, American Type Culture Collection, Manassas, VA, USA) was cultured in Trypticase Soy Broth (TSB: Becton Dickinson Biosciences, Franklin Lakes, NJ, USA) at 37°C for 6 hours to create a bacterial suspension of 7.5 × 10^7^ CFU/mL (logarithmic growth: Optical Density [OD] _600_ = 0.2; pH 7.0). Olson et al. investigated the superior adherence capability of PIA/PNAG-producing *S. epidermidis* on biomaterial surfaces [20]. In this research, we only used a PIA/PNAG-producing strain positive for the *icaA* gene as determined by RT-PCR [36]. Before the procedure, all test specimens were sterilized by way of ultrasonic cleaning and steam autoclaving. Two microliters of the bacterial suspension were dropped onto the specimens, which were then placed at room temperature for 60 minutes. The specimens were then rinsed twice with phosphate-buffered saline (PBS: Sigma-Aldrich St Louis, MO, USA; pH 7.0) to remove any unbound and deposited cells. The specimens were transferred into sterile conical tubes (Falcon®, BD Biosciences, Franklin Lakes, NJ, USA) with 5 mL of fresh TSB medium. The tubes were vortexed at full speed for 1 minute and then placed in an ultrasonic bath and sonicated for 15 minutes at 120 W to release the attached cells from the biomaterial. After an additional vortex step, the specimens were removed and the remaining suspensions were diluted with PBS and cultured at 37°C for 48 hours with a Compact Dry TC culture kit (Nissui Pharmaceutical Co., Ltd., Tokyo, Japan). Colony-forming units (CFUs) were counted to determine the number of viable adherent bacteria, and the bacterial density (CFU/ml) was calculated. The above procedure was performed twenty times for each material. As well as using uniform conditions for the bacteria, the experiments themselves were repeated using a uniform procedure to eliminate the effect of environmental factors such as temperature and pH.

### Statistical analysis

The means and standard deviations of the topographic parameters of the specimens (n = 6), contact angles (n = 12) and viable adherent bacteria densities (n = 20) were analyzed for each material in both groups using the *Mann-Whitney U test* with SPSS 10.0 statistical software (SPSS Inc., Chicago, IL, USA). Statistical analysis of the materials was performed using one-way analysis of variance (one-way ANOVA), multiple comparison tests and the Tukey-Kramere and Bonferroni/Dunn multiple comparison test for *post hoc* analysis. The value of statistical significance was set at *P* < 0.05.

## Results

**Figure 1:**
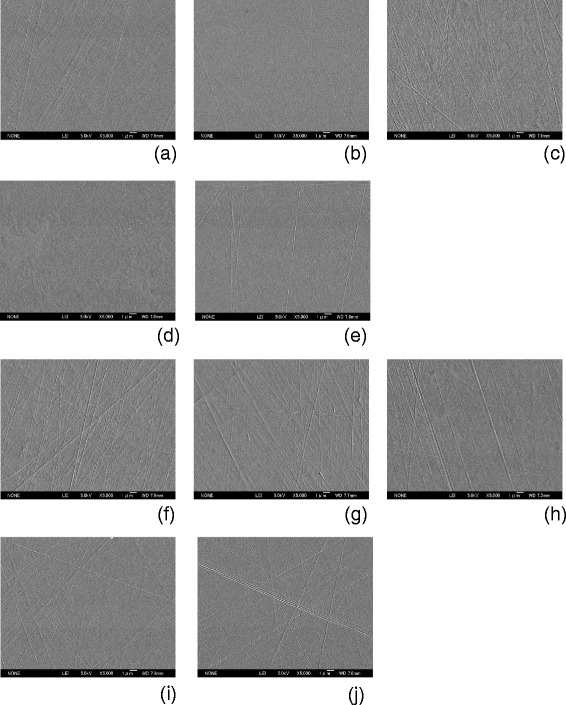
**SEM micrographs.** The fine group specimens had a relatively featureless surface compared to the coarse group specimens. Fine group: Oxinium **(a)**, Co-Cr-Mo **(b)**, Ti-6Al-4 V **(c)**, Cp-Ti **(d)**, SUS316L **(e)**. Coarse group: Oxinium **(f)**, Co-Cr-Mo **(g)**, Ti-6Al-4 V **(h)**, Cp-Ti **(i)**, SUS316L **(j)**. Original magnification × 5000 (Scale bar =1 μm).

**Figure 2:**
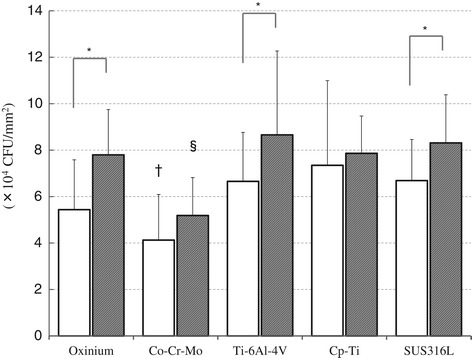
**Viable adhered cell count of** ***S. epidermidis*** **(×10** ^**5**^ **/mL).** Mean and standard deviation are shown. *: *P* < 0.05. †: *P* < 0.05 compared with Ti-6Al-4 V, Cp-Ti, or SUS316L. §: *P* < 0.05 compared with Oxinium, Ti-6Al-4 V, Cp-Ti, or SUS316L. □ Fine group, ■ Coarse group.

Field emission scanning electron microscope images of the prepared disk surfaces are shown in Figure 1. All specimens were observed to have micro-traces of polishing distributed over the surface, but this was more conspicuous in the coarse group. The mean surface roughness parameters for each type of specimen are shown in Table 1. In the fine group, all specimens had comparatively smooth surfaces and recorded low average roughness (Ra: 1.8-8.5 nm, <10 nm); however, the specimens in the coarse group exhibited comparatively rougher surfaces (Ra: 7.2-30.0 nm). Statistical analysis revealed that the differences in the Ra value between the two groups were statistically significant for all biomaterials. One-way ANOVA indicated some significant differences in Ra value among the various materials in both the fine group and the coarse group. The contact angles of deionized water are shown in Table 2. Generally, it is considered that the rougher surface can generate more hydrophobicity. However, there were no significant differences in water contact angles between the two groups except for Ti-6Al-4 V. Of the various materials, the surface of Co-Cr-Mo demonstrated the highest water contact angle in both groups. The results of the adhesion of *S. epidermidis* to both groups of the various specimens are shown in Figure 2. Larger amounts of *S. epidermidis* adhered to each specimen in the coarse group than in the fine group. In particular, Oxinium, Ti-6Al-4 V and SUS316L demonstrated statistically significant differences between the fine group and the coarse group (*P* < 0.05). The Co-Cr-Mo specimens in the fine group had significantly lower adherence than the Ti-6Al-4 V, Cp-Ti and SUS316L specimens (*P* < 0.05). Similarly, the Co-Cr-Mo specimens in the coarse group exhibited significantly lower amounts of adhered bacteria than the other four materials (*P* < 0.05).

**Table 1:** **Surface roughness**

	**Ra (nm)**		
	**Fine group**	**Coarse group**	**P-value**
Oxinium	8.5 (0.5)^b,d,e^	30.0 (2.0)^b,e^	0.004
Co-Cr-Mo	5.8 (0.2)^a,c,e^	12.0 (1.9)^a^	0.004
Ti-6Al-4 V	7.1 (0.4)^b,d,e^	16.5 (14.5)	0.003
Cp-Ti	5.6 (1.2)^a,c,e^	22.0 (6.0)	0.004
SUS316L	1.8 (0.4)^a,b,c,d^	7.2 (1.9)^a^	0.002

Data were expressed as a mean (standard deviation (SD)).

Ra: arithmetic mean of the departure of the roughness profile from the profile center-line.

^a^ *P* < 0.01 compared with Oxinium.

^b^ *P* < 0.01 compared with Co-Cr-Mo.

^c^ *P* < 0.01 compared with Ti-6Al-4 V.

^d^ *P* < 0.01 compared with Cp-Ti.

^e^ *P* < 0.01 compared with SUS316L.

**Table 2:** **Contact angles of deionized water (degree)**

	**Contact angle (degree)**		
	**Fine group**	**Coarse group**	**P-value**
Oxinium	73.9 (5.6)^b,d,e^	76.3(9.2) ^b,c,d,e^	0.33
Co-Cr-Mo	104.1 (5.7)^a,c,d,e^	105.8 (1.0) ^a,c,d,e^	0.06
Ti-6Al-4 V	77.0 (5.3)^b,d,e^	84.7 (3.0) ^a,b,e^	0.002
Cp-Ti	89.2 (7.1)^a,b,c^	84.8 (3.0) ^a,b^	0.20
SUS316L	90.0 (2.3) ^a,b,c^	91.2 (2.0) ^a,b,c^	0.39

Data were expressed as a mean (standard deviation (SD)). A greater water contact angle means a more hydrophobic surface. Oxinium had the smallest water contact angle, indicating the most hydrophilic surface.

^a^ *P* < 0.01 compared with Oxinium.

^b^ *P* < 0.01 compared with Co-Cr-Mo.

^c^ *P* < 0.01 compared with Ti-6Al-4 V.

^d^ *P* < 0.01 compared with Cp-Ti.

^e^ *P* < 0.01 compared with SUS316L.

## Discussion

In this *in vitro* study, we compared the bacterial adherence capability of PIA/PNAG-positive *S. epidermidis*, which is the preeminent cause of implant-related infection, on five types of biomaterials, investigating substratum surface roughness at different levels of roughness below 30 nm Ra. Defining the minimum level of roughness at which bacterial adhesion occurs can provide useful findings about the mechanism of the early stages of implant-related infection.

The duration of adherence without any formation of biofilm was set for 60 minutes, because the strain used in this experience had a high level of adherence capability [36]. Therefore, the results can confidently be regarded as early adhesion. There is little risk of the suspension evaporating, possibly because of the relatively high air humidity in Japan. Consequently, we did not need additional TSB for the incubation period. Since contamination during surgery is thought to be the main cause of implant-related infection, early adhesion ability during the several minutes or hours between the removal of the implant from its package and its implantation is clinically important.

The results of this study indicate that there were statistically significant differences in the total amount of viable bacteria that adhered to Oxinium, Ti-6Al-4 V and SUS316L between the fine group and the coarse group. Research has highlighted a particularly positive correlation between early bacterial adhesion and surface roughness [28-31]. Surface roughness not only increases the surface area for bacterial adhesion, but is also thought to provide a scaffold that facilitates bacterial adhesion. Taylor et al. reported that a small increase in the roughness of PMMA (Ra = 1.24 μm) resulted in a significant increase in bacterial adhesion over the smoother PMMA surface (Ra = 0.04 μm) [37]. Quirynen et al have reported that *in vivo* surface roughness below 0.2 μm (200 nm) Ra does not affect bacterial adhesion [32,33]. Lee et al demonstrated no significant difference in bacterial adherence capability between titanium (Ra = 0.059 μm) and zirconia (Ra = 0.064 μm), but significantly high amounts of bacteria adhered to resin (Ra = 0.179 μm) [34]. However, Öztürk et al indicated that a difference in roughness of 3 to 12 nm Ra between as-polished and nitrogen ion-implanted Co-Cr-Mo contributes to bacterial adhesion behavior [35]. The cause of this non-linear dependence and discordance in the previous studies concerning bacterial adhesion on surface roughness poses a question about the minimum level of surface roughness. As clinically different prostheses or implant devices have different [degrees of] surface roughness that may play a role in bacterial adhesion and implant infection, it is necessary to evaluate bacterial adherence capability on the same kind of original materials over quite a low range of surface roughness in order to define the minimum threshold.

The results of our *in vitro* research suggest that even quite a low surface roughness range of 8.5-30.0 nm Ra for Oxinium, 7.1-16.5 nm Ra for Ti-6Al-4 V and 1.8-7.2 nm Ra for SUS316L can influence bacterial adhesion (*P* < 0.05). These findings concur with Öztürk et al [35]. The nanometer scale of roughness on the deposition of micron-sized bacteria may be associated with structures on the cell surface much smaller in size than the organisms themselves, i.e. flagella, lipopolysaccharides or extracellular polymeric substances. At the same time, it may also suffice to say that the surface roughness range of 5.8 to 12.0 nm Ra for Co-Cr-Mo and 5.6 to 22.0 nm Ra for Cp-Ti did not demonstrate a statistically significant difference for *S. epidermidis* adhesion in this study. These results indicate that the minimum level of roughness required for *S. epidermidis* adhesion differs according to the type of biomaterial used, and that adhesion is a multi-factorial process that is unlikely to be explained by a single surface characteristic.

Among the materials in both the fine and coarse groups, adherence was significantly lower for the Co-Cr-Mo specimens than for the Ti-6Al-4 V, Cp-Ti and SUS316L specimens (*P* < 0.05). Needless to say, Ti-6Al-4 V, Cp-Ti and SUS316L have high biocompatibility, and therefore are considered to provide more favorable surfaces for bacterial adherence. When comparing the surface roughness in each group, it is difficult to say whether the degree of bacterial adhesion was affected by surface roughness alone. In particular, SUS316L showed a similar or even higher degree of adhered *S. epidermidis* compared to the other biomaterials despite having the lowest surface roughness in each group. Surface wettability (water contact angle) is another crucial element influencing bacterial adhesion [24,26,29,32]. Boks et al reported that bond strengthening for four strains of *S. epidermidis* on a hydrophobic surface was fast and limited to a minor increase, while the strengthening of bonds on a hydrophilic surface increases significantly with contact time [38]. Tang et al concluded that on the hydrophobic surface there were fewer adhered bacteria and they did not clump together readily [39]. As water molecules adjacent to a hydrophobic surface are not able to form hydrogen bonds with that surface (hydrophobic effect), bacterial adhesion to a hydrophobic specimen is brought about by an entropically favorable release of water molecules. The results of this research indicated that the amount of bacteria that adhered to the more hydrophobic Co-Cr-Mo surface was significantly less than that of the more hydrophilic materials. However, Tegoulia et al found that a hydrophilic surface provides a stable interfacial water layer and prevents direct contact between the bacteria and the surface [40]. Concerning Ti-6Al-4 V in our study, although the coarse group exhibited more hydrophobicity than the fine group, more bacterial adhesion was observed. Therefore, it is possible that bacterial adhesion is a multi-factorial phenomenon, and surface roughness and wettability are not the only material surface characteristics influencing *Staphylococcal* adherence. Further study is needed to refine the difference in bacterial adherence capability among the different types of biomaterials.

Several *in vitro* and *in vivo* studies found low bacterial adhesion on zirconia ceramics, which are compositionally similar but not identical to Oxinium [41,42]. Poortinga et al. showed that the change in substratum potential as a function of the number of adherent bacteria is a measure of the amount of electric charge transferred between the substratum and the bacteria during adhesion [43]. With Oxinium having a ceramic surface, it was thought that the electron transfer or electrical potential may be different from the other four metallic biomaterials. However, Oxinium in this study exhibited no statistical suppression of the amount of adhered bacteria compared to the other materials (*P* > 0.05).

Several limitations must be noted in interpreting the data. The pathogenesis of prosthetic device infections is a complex process involving interactions between the pathogen, the biomaterial and the host. An *in vitro* study cannot account for host defense and other *in vivo* factors such as temperature, flow conditions and nutrition. However, the results of our *in vitro* research suggest a lower degree of adhesion of *S. epidermidis* to Oxinium, Ti-6Al-4 V and SUS316L in the fine group than in the coarse group, which indicates the minimum level of roughness required for bacterial adhesion, as well as low adhesion to the relatively hydrophobic Co-Cr-Mo. As the next stage of this research, we need to assess the detailed mechanisms of bacterial adhesion under more sophisticated conditions. This study allowed greater control of the experimental variables and produced fewer artifacts in the results. Although the complex phenomena that occur *in vivo* could not be accurately reproduced*,* it was possible to make a simple comparison of bacterial adhesion capability on various material surfaces of different roughness that are actually used in clinical practice. We consider that our study has provided valuable results regarding the early stages of assessment of implant-related infection. These simple configurations are particularly encouraging as tests for use.

## Conclusions

We compared the adherence capability of *S. epidermidis* to surfaces at different levels of roughness below 30 nm Ra using five types of solid biomaterials. The total amount of viable bacteria that adhered to Oxinium, Ti-6Al-4 V and SUS316L was significantly greater in the coarse group than in the fine group. Co-Cr-Mo, which has more hydrophobic surface, demonstrated less bacterial adherence than the other materials.
